# Solid tumor rejection using personalized incompatible human leukocyte antigen (HLA) and blood group ABH antigens

**DOI:** 10.37349/emed.2025.1001375

**Published:** 2025-11-28

**Authors:** Apostolos P. Georgopoulos, Lisa M. James

**Affiliations:** 1 The HLA Cancer Research Group, Brain Sciences Center, Department of Veterans Affairs Health Care System, Minneapolis, MN 55417, USA; 2 Department of Neuroscience, University of Minnesota Medical School, Minneapolis, MN 55455, USA; 3 Institute for Health Informatics, University of Minnesota Medical School, Minneapolis, MN 55455, USA; 4 Department of Psychiatry, University of Minnesota Medical School, Minneapolis, MN 55455, USA

**Keywords:** cancer, immunotherapy, human leukocyte antigen (HLA), antibodies, vaccines, blood groups

## Abstract

Cancer immunotherapies have become mainstream, targeting tumor elimination via various mechanisms, albeit with varied effectiveness. Here, we review briefly the current landscape of cancer immunotherapies and the central role of human leukocyte antigen (HLA) in them. We then propose a new kind of immunotherapy for solid tumors, where the key is the involvement of antigens and antibodies unrelated to the tumor itself. In this approach, we consider the tumor as akin to a transplanted organ, which can be rejected by two different mechanisms of incompatibility. The first involves the intra-tumor administration of mRNA blueprints of incompatible (to the patient) HLA proteins, leading to their synthesis and, hopefully, elicitation of an anti-tumor immune reaction, assuming immunocompetency. The second therapy involves the direct, intra-tumor administration of anti-A/B/H blood group antibodies lining the blood and lymph vessels of the tumor. In organ transplantation, AB incompatibility leads to organ rejection, and the same effect would be expected when anti-A/B/H antibodies (depending on the patient’s ABO group) are injected into the tumor. Notably, the anti-tumor effect by the preformed anti-blood group antibodies is complement-mediated and should not be affected by tumor immunoevasion. This proposed cancer immunotherapy aimed at promoting tumor rejection via antigen incompatibility offers a novel cancer treatment approach that warrants further investigation.

## Graphical Abstract

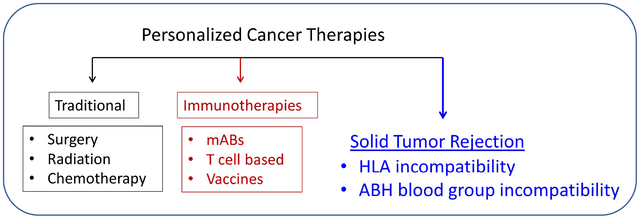

**Schematic diagram of the outline of the review.** HLA: human leukocyte antigen.

## Introduction

There are many different cancer therapies, including surgery, chemotherapy, radiation, and, more recently, immunotherapy. Although significant advances have been made, cancer often remains a fatal disease. With respect to older, standard treatments (surgery, chemotherapy, radiation), cancer therapy has always been personalized, in the sense that the choice and application of a specific treatment has been based on the joint evaluation of the nature of the cancer (e.g., histological type), location and stage, the presence of metastases, and the condition of the patient. Beyond this simple notion of “personalized” therapy, more specific applications of personalized/precision cancer therapies include the use of multi-omics data from the tumor and the patient to optimize treatment and predict its outcome [1] and cancer immunotherapy [2]. The first monoclonal antibody (rituximab) for the treatment of B-cell lymphoma was approved by the U.S. Food and Drug Administration (FDA) in 1997. This marked the beginning of the era of cancer immunotherapy, which is currently at the mainstream of cancer therapies. At the center of cancer immunotherapies lies the human leukocyte antigen (HLA), namely the immunogenetic makeup of the patient [3]. Here, we review briefly the basics of HLA and various cancer immunotherapies, and propose a new approach to personalized cancer therapy based on induced incompatibility of HLA and ABH blood group antigens between the tumor and the patient, essentially treating the tumor as a foreign tissue to be rejected.

## Human leukocyte antigen (HLA)

### General

HLA codes for cell-surface glycoproteins that present antigens to T cells for protection of the host against peptide antigens emanating from non-self proteins. There are 2 HLA classes (I and II), each of which comprises 3 classical genes: *A*, *B*, *C* (Class I) and *DPB1*, *DQB1*, *DRB1* (Class II). Each gene codes for a number of specific proteins. In the current 2-field notation, an HLA molecule is denoted by a string of 3 parts comprising the gene, the allele group, and the protein sequence coded by a specific allele. For example, HLA-A*11:15 denotes a unique protein (amino acid sequence) coded by the variant A*15:11 of gene A and allele group 15 of HLA Class I. A given gene codes for many allele groups, and each allele group codes for many different unique proteins, so that there exists a very large number of HLA-coded proteins [4].

HLA-I molecules are expressed in all nucleated cells and bind peptides coming from partial degradation of endogenous proteins (e.g., rapidly degraded self proteins, proteins of pathogens, tumor proteins, etc.) in the proteasome. By contrast, HLA-II molecules are typically expressed in the professional antigen-presenting cells (macrophages, dendritic, and B cells), but also in other cell types [5], including cancer cells [6–8]. HLA-II molecules bind peptides coming from partial degradation of exogenous proteins (internalized by pinocytosis and/or phagocytosis) in the lysosomal system. In addition, to some extent, peptides from proteins from either source (endogenous, exogenous) are made available to HLA-I or HLA-II molecules via the cross-presentation system [9]. Detailed reviews of HLA function and antigen processing and presentation can be found elsewhere [10–12].

### Peptide binding and presentation

HLA-I molecules typically bind to peptides of 8–10 amino acid length (mostly 9-mers), whereas HLA-II molecules typically bind peptides of 13–18 amino acid length (mostly 15-mers). HLA molecules of both classes possess very high specificity and degeneracy [13, 14]. With respect to the former, a specific HLA molecule can bind with high affinity to only a few non-self antigens among a large number available, and can distinguish peptide antigens by even single amino acid substitutions. And with respect to the latter, a particular HLA molecule can bind a large number of peptides.

HLA-I and HLA-II molecules are unstable without a bound peptide and very stable when bound to a peptide with high affinity. In fact, the degree of stability is directly related to the strength of binding affinity. In the absence of a suitable peptide, HLA-I molecules are bound with low affinity to self peptides [15]. When a high-affinity peptide appears, the self peptide is replaced by it, conferring stability and paving the way for the peptide-HLA-I (pHLA-I) complex to move to the cell surface and present its peptide to T lymphocytes (T cells) that express the CD8 coreceptor (CD8+ T cells). Similarly, HLA-II molecules are also unstable without a bound peptide. In the absence of a high-affinity peptide, they are loosely bound to a local peptide, invariant chain (Ii) [16]. When a high-affinity peptide appears, it replaces Ii, the pHLA-II molecule thus formed is very stable and moves to the surface to present its peptide to T cells that express the CD4 coreceptor (CD4+ T cells). The stability of the pHLA-I and pHLA-II complexes is very important because it determines the time for which bound peptides can be presented to T cells: the longer they are presented, the higher the probability of attracting a T cell and the higher the number of T cells activated, amounting to a higher level of T cell activation.

### Recent advances

Several advances in antigen processing and presentation have been made in recent years in diverse fields, including imaging of the pHLA complex [17], origin of peptides presented to HLA molecules [18], peptide transport and presentation [19], and pHLA dissemination by extracellular vesicles (EV) [20], among others. We review these advances briefly below.

#### Imaging

The current status and progress in the field of imaging in vitro and in vivo processes involved in antigen processing, presentation to HLA molecules, and T cell activation have been reviewed comprehensively [17]. The development of many diverse methods for imaging of cell immune processing is impressive, including the use of special tracers for real-time monitoring of enzyme activity in the proteasome [21, 22], endoplasmic reticulum [23], and endolysosomal compartment [24]; the use of positron emission tomography (PET) for visualizing HLA-II activation (Immuno-PET) [25]; and the use of various combined techniques to monitor T cell activation [26–28], to mention but a few. It is expected that the integration of the data obtained from this fast-moving field with that obtained by parallel advances in other technologies would yield almost qualitatively new understandings of the real-time processing of cellular immune functions.

#### Origin of peptides presented to HLA molecules

The immunopeptidome is the ensemble of peptides available for binding to HLA-I and HLA-II molecules [29]. The peptides in the immunopeptidome come from various sources, including endogenous proteins and exogenous proteins (by limited proteolysis in the proteasome and lysosomal compartment, respectively), and free polypeptides derived from defective ribosomal products (DRiPs) [30]. A recent addition to the immunopeptidome are peptides derived by translating non-coding RNA (ncRNA) [31, 32], including long ncRNA (lncRNA) and circular RNA (cRNA) [18], which can be translated to peptides [33], e.g., via internal ribosome entry sites [34, 35]. Such peptides can bind to HLA molecules, are immunogenic, and can play an important role against the tumor [32]. Finally, post-translationally modified proteins (PTMPs) also provide a rich source of peptides to the immunopeptidome [36].

#### Peptide transport and presentation

Ordinarily, in the HLA-I system, short peptides (8–10 mers) are transported from the cytosol to the lumen of the endoplasmic reticulum by the transporter associated with antigen processing (TAP) [37], where they bind to HLA-I molecules. Excluding a bound peptide, these molecules consist of a conserved soluble serum protein β_2_-microglobulin (β_2_m) and a polymorphic α heavy chain [38, 39]. Traditionally, TAP and β_2_m have been considered necessary for peptide binding to HLA-I molecules. A recent study [19] has documented the successful binding of peptides to HLA-I molecules, followed by presentation to, and successful activation of, T cells in the absence of TAP or β_2_m. In TAP-null cells, evidence indicates that peptides are transported to the HLA-I molecule by the Se62 protein, whereas in β_2_m-null cells, a successful binding could be supported by a suitable conformational change in the heavy α chains of the HLA-I molecule [19]. Given that TAP and β_2_m expression are frequently depressed in cancer (see below), these findings indicate that immunotherapy based on CD8+ T cell activation could still be effective even in these cases.

#### pHLA dissemination by EVs

Traditionally, the presentation of peptides to T cells has been regarded as a local affair, in the sense that this presentation and T cell engagement take place in the locale of the presenting cell. For example, pHLA-I complexes stay on the cell surface for a period of time and then are internalized and recycled [40]. Although this is true, a number of these complexes are released from the cell surface in a soluble form and transported to other locations via EVs [20, 41, 42]. This mechanism allows for distant dissemination of diverse immune-related material (e.g., peptides of the immunopeptidome) with a number of potential therapeutic applications in cancer therapy [18].

### CD8+ and CD4+ T lymphocytes

Peptides presented by the HLA-I and HLA-II molecules bind to the T cell receptor (TCR). Although the same generic notation (TCR) is used for both HLA molecules, the biophysics are different in the two cases, since two different, T cell specific, coreceptors are involved, namely CD8 for the pHLA-I specific CD8+ T cells and CD4 for the pHLA-II specific CD4+ T cells. Both coreceptors increase the stability of the pHLA complex and facilitate the ultimate activation of the T cell for an effective discharge of their effector actions described below. The peptide-TCR binding is characterized by very high specificity, sensitivity, and degeneracy. With respect to specificity, even a single amino acid residue substitution in the peptide can make a big difference in the probability of its binding to TCR, for both pHLA-I and pHLA-II molecules. With respect to sensitivity, a CD8+ T cell can be activated by a single pHLA-I complex [43], and a CD4+ T cell can be activated by a single pHLA-II complex [44]. And with respect to degeneracy, a single TCR of a CD8+ or CD4+ cell can be engaged by many pHLA complexes.

The main function of CD8+ T cells is the elimination of the cell that contains the peptide (antigen) presented by the pHLA-I complex. This is achieved by (a) direct cytotoxicity via the perforin-granzyme and Fas/FasL pathways leading to cell death (apoptosis), (b) the production and secretion of various cytokines enhancing the elimination and clearance of the target cell, mainly interferon gamma (IFN-γ) and tumor necrosis factor alpha (TNF-α), and (c) other mechanisms (e.g., bystander killing, pyroptosis, ferroptosis).

The activation of CD4+ T cells promotes and facilitates the elimination of non-self target cells by various mechanisms, including (a) the initiation of the production of antibodies by B cells against the presented peptide (and hence its associated protein), (b) enhancement of the function of cytotoxic CD8+ T cells, (c) production and secretion of various cytokines (e.g., IFN-γ) recruiting and enhancing the function of other cells (e.g., macrophages) aiding in the elimination and clearance of the target cell, and (d) direct cytotoxicity.

Following exposure to, and activation by, suitable antigens presented by pHLA-I and pHLA-II complexes, subsets of both CD8+ and CD4+ T cells differentiate into “memory cells” that react fast and effectively when those non-self antigens (e.g., from pathogens) are encountered in the future. Memory T cells and antibodies provide a timely, effective, and efficient response to eliminate previously presented non-self antigens (mostly viruses) when they reappear in the future.

### HLA in cancer: immunoevasion

In cancer, HLA function is commonly suppressed, resulting in “immunoevasion”. Suppression of HLA-I expression has been well documented in various cancers due to various reasons [45–51]. In addition to HLA-I suppression, the function of antitumor cytolytic CD8+ T cells (the activation target of pHLA-I) is also suppressed [52]. The overall result of HLA-I suppression and CD8+ T cell exhaustion is promotion of tumor growth. Cytolytic T cell antitumor activity can be partially restored by immune checkpoint inhibitors (ICIs) therapy [53], but HLA-I suppression due to genetic alterations cannot be restored. Finally, it should be noted that HLA-I/CD8+ T cell dysfunction would adversely affect an otherwise positive outcome of the peptide-based cancer vaccines (discussed below), a therapy that would profit from a concomitant ICI therapy, as has been observed [54].

As mentioned above, HLA-II molecules are normally expressed mainly in professional antigen-presenting cells, but they have also been detected in other tissues [5] and cancer cells [6–8]. Alterations in the expression of HLA-II molecules in cancer cells have been observed in various tumors, intensities, and types affecting different stages in the biosynthesis of HLA-II molecules and their regulation [55–57]. Overall therapeutic effects have been associated with HLA-II expression in various cancers, especially with an enhanced effect of concomitant ICI treatment [8, 58–62].

## Cancer immunotherapy

In addition to traditional treatments for cancer (e.g., surgery, radiation, chemotherapy, etc.), during the past several decades, there have been tremendous advances in cancer treatments, spurred by developments in cancer immunotherapy. As a result, cancer survival rates have markedly improved; nonetheless, a substantial number of cancer patients are resistant to treatment, experience cancer re-emergence, or experience significant adverse effects, all of which contribute to cancer mortality. Consequently, calls for personalized cancer treatment approaches have grown louder. Moreover, it is increasingly recognized that novel treatment strategies are warranted. Here, we provide a brief overview of existing cancer immunotherapies and introduce novel personalized treatment approaches for which HLA takes center stage.

Cancer immunotherapy rests on harnessing an individual’s immune system to more effectively fight cancer. Under optimal conditions, immune surveillance permits recognition of tumor antigens, prompting T-cell-mediated elimination of tumor cells, a process that is critically dependent on HLA (also known as major histocompatibility complex, MHC) as described below. Tumor cells, however, are notorious for escaping immune surveillance and/or immune response and altering the tumor microenvironment in favor of cancer proliferation. Cancer immunotherapy aims to enhance recognition of tumor antigens and stimulate T cell activity aimed at the elimination of cancer cells.

### Tumor antigens

Tumor antigens can be classified as tumor-associated antigens (TAAs) or tumor-specific antigens (TSAs) [63]. TAAs are preferentially found or are overexpressed in tumors but may also be present in other cells; they include differentiation antigens and cancer germline antigens/cancer testis antigens (CTAs). CTAs are ideal targets for immunotherapy; they are highly immunogenic and, while overly expressed in various tumors, their expression in normal tissue is limited to the testis and placenta, which are immunologically privileged, thus reducing the likelihood of adverse autoimmune responses [64, 65]. Indeed, CTAs such as melanoma antigen gene (MAGE) and New York esophageal squamous cell carcinoma oncoprotein 1 (NY-ESO-1) proteins have been the targets of numerous immunotherapy trials [66, 67]. Human epidermal growth factor receptor 2 (HER2), a protein associated with the rapid growth of breast cancer, is another TAA for which targeted immunotherapies have been particularly effective, albeit with high rates of relapse or disease progression [68]. In contrast to TAAs, which are expressed on normal tissues, TSAs are uniquely found on tumor cells. TSAs include mutated proteins commonly referred to as neoantigens and viral antigens such as human papilloma virus (HPV), Epstein-Barr virus (EBV), and human endogenous retroviruses (HERVs) (e.g., viral antigens, neoantigens) [63]. Because they are not expressed in normal tissues [69] and escape negative selection as non-self antigens [70], TSAs—particularly, neoantigens [71–73]—have increasingly become immunotherapy targets. The meteoric growth of interest in neoantigen-based cancer immunotherapy studies has led to promising advancements in personalized cancer immunotherapy [74, 75] and identified limitations of neoantigen-based immunotherapeutic approaches, including high neoantigen heterogeneity even within a given tumor [49] with relatively few neoantigens that are highly immunogenic [72, 76–80], potentially contributing to immune escape. Ongoing efforts are aimed at the identification of immunogenic epitopes and optimizing strategies to target both TAAs and TSAs via various types of immunotherapies [74, 80, 81].

In general, cancer immunotherapies share a common goal of enhancing the immune system’s anticancer activity, albeit they operate via different strategies. Standard immunotherapeutic strategies, including monoclonal antibodies (mAbs), T cell therapies, and cancer vaccines, reviewed elsewhere [82, 83], are summarized below.

### mAbs

Briefly, mAbs are antibodies that are manufactured to bind to specific target antigens (e.g., HER2, immune checkpoints), causing tumor cell death by various effector mechanisms [68, 84]. Dozens of mAbs are FDA-approved and used clinically to treat cancer [83]. ICIs, a specific type of mAbs that bind with checkpoint proteins that would otherwise suppress the ability of T cells to destroy a tumor, are the first or second line of treatment for several cancers [82, 85]. Treatment with mAbs has been shown to significantly improve survival [82, 84, 86, 87]; however, serious side effects, resistance to treatment, and cancer re-occurrence are common limitations of mAbs [85, 88–90].

### T cell therapies

T cell therapies involve collecting and manipulating a patient’s T cells to more effectively recognize and kill cancer cells. T cell therapies for cancer include infusion of tumor-infiltrating lymphocytes (TILs) obtained from a resected tumor and grown in large quantities ex vivo or infusion of T cells that are genetically engineered to express either a synthetic chimeric antigen receptor (CAR) that recognizes cell-surface tumor antigens or a T-cell receptor (TCR-T therapy) that recognizes intracellular tumor antigens [67]. Serious adverse effects, poor persistence, cancer cell resistance, and prohibitive costs limit the use of CAR-T cell therapies primarily to hematological cancers [67, 91, 92]. Similarly, toxicity and cross-reactivity with normal tissues are a concern for TCR-T [93], although it is relatively safer than CAR-T because of its MHC restriction [94] and has proven at least somewhat beneficial for synovial sarcoma and melanoma (targeting CTAs) and for HPV-associated cancers (targeting HPV antigens) [67]. As far as safety, TIL therapy has the advantage as it relies on a patient’s natural T cells [83]; however, costly and time-consuming production limits their use to the second line of therapy or adjunctive to ICIs [95].

## Cancer vaccines

### Tumor antigen-based vaccines

Therapeutic cancer vaccines stimulate T cell responses to target TAAs or TSAs via various platforms, including dendritic cells (DCs), viruses, peptides, and nucleic acids, as reviewed elsewhere [66, 96–99]. Cancer vaccines are safe, and several have been approved for use [100–102]. In recent years, peptide-based and nucleic acid-based vaccines have attracted substantial attention [103–106]. Peptide-based vaccines present tumor antigen epitopes to antigen-presenting cells, where the epitopes bind with MHC molecules and are presented to T cells to facilitate elimination of tumor cells. Although an appealing and promising approach to cancer immunotherapy, peptide-based cancer vaccine trials to date have demonstrated limited efficacy [104]; however, identification of optimal antigens to enhance immune response against tumor cells is an active area of investigation and a strategy that is ripe for personalized interventions [98, 104, 105]. The recent development of nucleic acid-based vaccines has ushered in new approaches for cancer treatment. The basis of nucleic acid-based vaccines for cancer rests on delivering genetic instructions for the production of cancer antigens, which once manufactured, are bound to HLA molecules, signaling cell destruction [105, 106]. As with peptide-based vaccines, selection of immunogenic antigens is a crucial factor in the effectiveness of nucleic acid-based vaccines. Immunogenicity, at least in part, is determined by individual variation in HLA; thus, individual HLA composition is at the very core of personalized cancer immunotherapy. In fact, to achieve the goal of eliminating a cancer (or infected) cell, the single necessary and sufficient step for T cell activation is the formation of a stable pHLA complex, a condition that rests on the specific HLA alleles carried by the patient. Advances in bioinformatics and sequencing permit the prediction of immunogenicity that can be leveraged to identify optimal antigens.

### HLA-based vaccines

As reviewed above, a major effort in tumor elimination is HLA-dependent, in that immunogenic peptides of TAA are delivered to the tumor, systemically or via DC pulsing, to maximize their binding to HLA-I molecules, leading to activation of CD8+ T cells and ensuing killing of tumor cells by the mechanisms mentioned above. This approach is usually more effective with concurrent administration of ICI treatment. The essential requirement for a successful outcome is that the peptides administered bind with high affinity to at least some of the six HLA-I molecules that a given patient carries. In most studies, the selection of the administered peptides is based on general considerations of their antigenicity but, as discussed above, the key lies in the strength of binding affinity of these peptides to the specific HLA-I molecules of the patient, a point taken seriously into account in some studies [107, 108].

A complementary alternative would be to enable the synthesis in the tumor cells of HLA-I molecules with predicted high-affinity binding to TAA epitopes [109]. In a recent study, we identified 9 HLA-I molecules with highly immunogenic binding to epitopes from 11 melanoma-associated antigens [110]. Such molecules could be synthesized from their mRNA blueprint and injected intratumorally. The same approach can be applied with respect to HLA-II molecules. In this case, the engagement of CD4+ T cells would have the dual advantage of (a) enhancing the cytolytic action of CD8+ T cells and (b) leading to the production of antitumor antibodies by B cells. To our knowledge, these approaches have not been tried yet.

## Tumor rejection

### Tumor rejection by antibodies against non-tumor antigens

All current cancer immunotherapies are directed against cancer cells. Here, we propose a new kind of immunotherapy that is also directed against the tumor itself, but through antibodies against non-tumor antigens. Such antigens may already exist naturally in the body (e.g., the ABH blood group antigens), or they can be synthesized by the tumor cells by injecting into the tumor relevant mRNA blueprints of these antigens (e.g., HLA molecules). In both cases, the destruction of the tumor is expected to resemble the rejection of a transplanted organ to an antigen-incompatible recipient. For example, without special pre-surgery preparation, organ transplants between ABO or HLA incompatible individuals are rejected at various times, from minutes or hours (“hyperacute rejection”, typically due to ABO incompatibility) to weeks or months (“acute rejection”, typically due to HLA incompatibility). In what follows, we discuss this approach, namely, treating a tumor as a foreign organ to be rejected.

### Tumor rejection due to incompatible HLA

In the case of organ transplantation, HLA proteins of the donor, expressed in the organ transplant (e.g., kidney), are highly immunogenic for a recipient that lacks them (“HLA incompatibility”). They are treated by the transplant recipient as non-self antigens, and, as such, are targeted for elimination by the usual immune mechanisms implemented by CD8+ and CD4+ T cells, resulting in destruction of the tissue that contains them and thus rejection of the transplant. In fact, HLA incompatibility accounts for most organ rejections [111, 112], hence the effort to match the HLA makeup of the recipient to that of the donor. Unfortunately, only monozygotic twins have a perfect match, and, otherwise, the closer (i.e., less incompatible) the HLAs of the donor and recipient are, the better the chance of transplant retention [111, 112].

**Figure 1.**
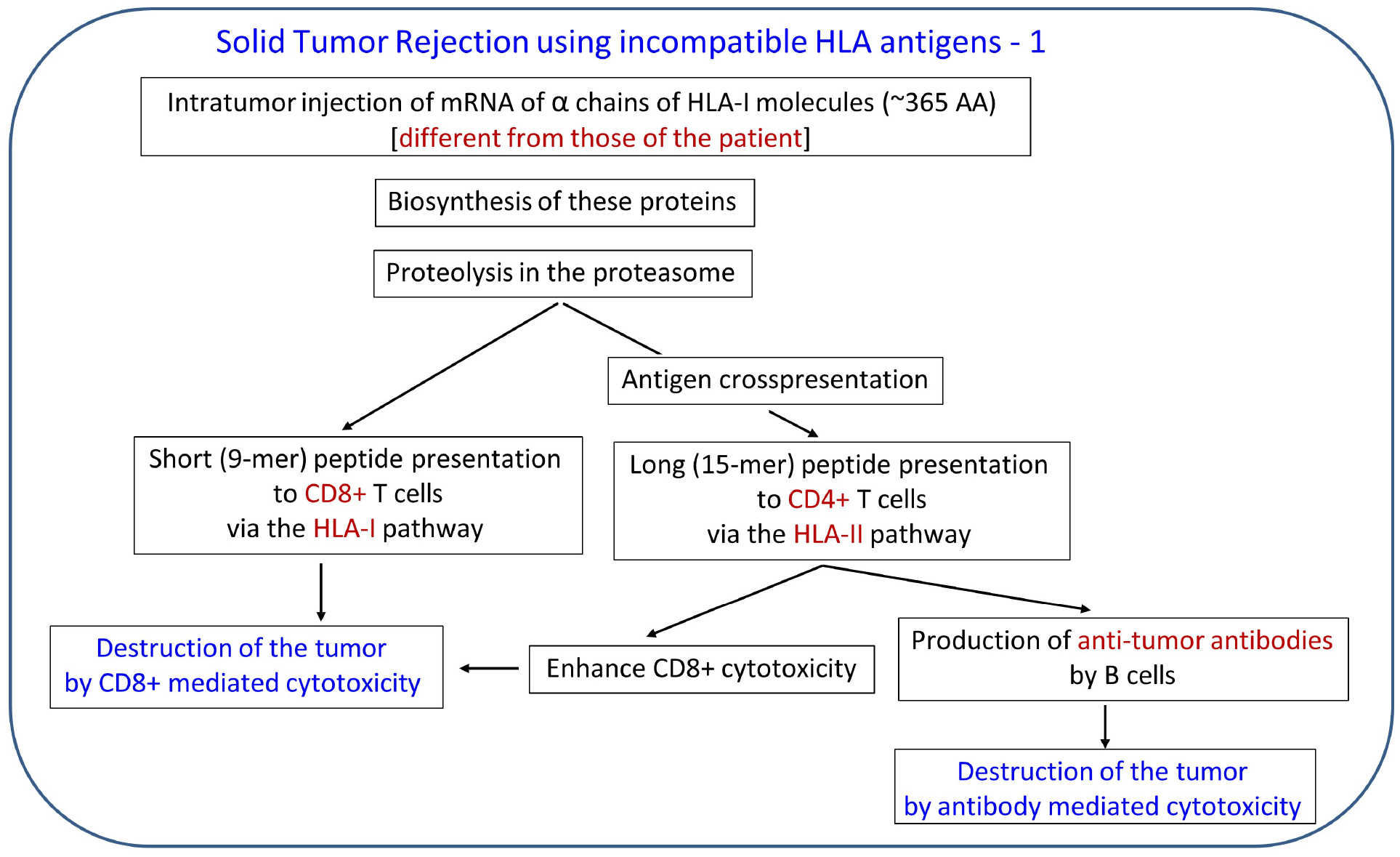
Outline of tumor rejection therapy based on HLA incompatibility. HLA: human leukocyte antigen.

**Figure 2.**
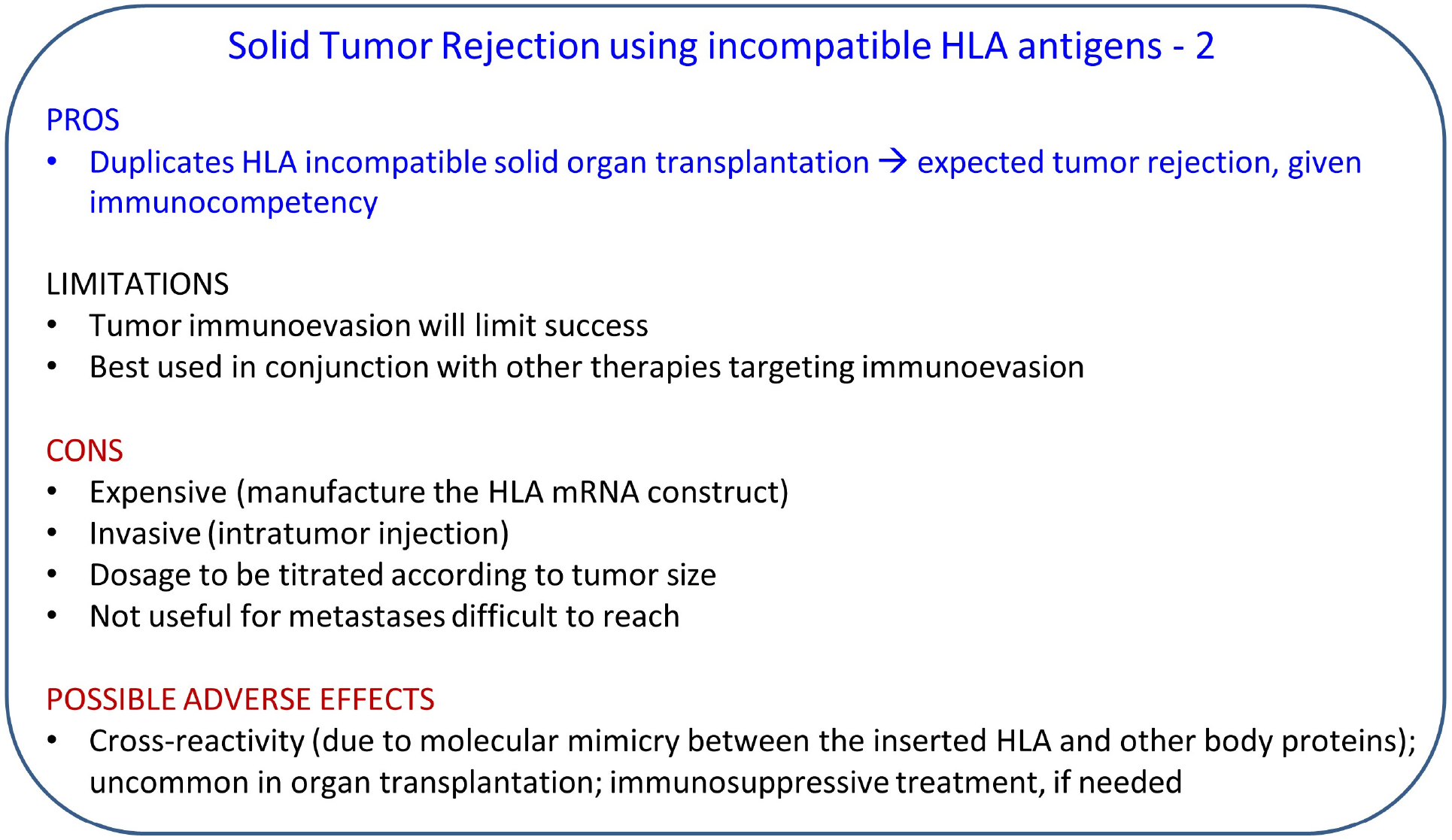
Features of tumor rejection therapy based on HLA incompatibility. HLA: human leukocyte antigen.

By contrast, in the case of cancer, the objective is just the opposite, namely, to reject the tumor. To that end, highly immunogenic proteins need to be introduced into the tumor, and HLA proteins fulfill that requirement, since they are known to be highly immunogenic. A realistic scenario for such an HLA-based approach for solid tumor rejection is outlined in Figures 1 and 2. In that treatment, HLA molecules, incompatible with those of the patient, would be introduced into the tumor (e.g., using their mRNA blueprint), essentially transforming the tumor to an incompatible transplant and leading to its destruction/rejection by the same mechanisms involved in rejecting an HLA-incompatible transplant. Proteins of the heavy α chain of HLA-I molecules would be ideal to use due to their known high immunogenicity, medium size (~365 amino acids), and available sequence(s); mRNA of several of them could be injected into the tumor. Given that the only requirement would be that they would differ (i.e., be incompatible) with regard to the HLA-I molecules of the patient, mRNAs of a large number of such proteins could be made, stored, and used without any delay in the case of a particular patient, a promising feature.

Now, although rejection of an HLA-incompatible transplanted organ is predictable and certain (“acute rejection”) without immunosuppressive treatment, in the case of a solid tumor, the outcome would depend on the status of the immune function/dysfunction at the tumor’s microenvironment (immunoevasion). For example, if the expression of the HLA-I/HLA-II molecules is suppressed or impaired, epitopes of the incompatible HLA molecule(s) inserted in the tumor (via their mRNA) may not be adequately presented to CD8+ and CD4+ T cells, the function of which might be impaired as well. Therefore, the outcome of this treatment could be quite variable and would depend on the extent to which CD8+/CD4+ T cell function has been restored by concomitant immunotherapy, such as ICI therapy.

With respect to potential adverse effects, it is possible that antibodies against the incompatible HLA molecules may attack other proteins in the body, mostly due to shared epitopes between the HLA inserted in the tumor and other body proteins (molecular mimicry). This occurs infrequently in solid organ transplantation. In fact, it was estimated that HLA-I proteins share < 0.8% epitopes with proteins of the human proteome [113]. These, and possibly other, considerations lead us to propose an approach that would involve direct antigen-antibody reaction against universal, preexisting antigens in the tumor, without the need to engage the immune system of the patient. This approach involves the blood group ABH antigens and the antibodies against them, as discussed next.

### Tumor rejection due to incompatible ABH blood group antigens

#### ABH human blood group antigens

There are two AB blood group antigens (A and B). They are cell-surface glycoproteins, formed by glycosyltransferases, enzymes coded by alleles A and B, respectively, at the ABO locus of chromosome 9 (9q34–1 and 9q34–2). These enzymes add specific sugars to the parent H antigen, thus producing antigen A by adding N-acetyl-galactosamine (GalNAc) at the end of an oligosaccharide chain, and antigen B by adding galactose (Gal) at the same position. Allele O encodes an inactive glycotransferase, leaving unmodified the presence of antigen H. ABH antigens are expressed in the membranes of red blood cells, platelets, endothelial cells, and epithelial cells [114, 115]. The 4 possible combinations of the presence or absence of the ABO antigens in an individual yield the 4 known ABO blood group types (A, B, AB, O), where the letter denotes the presence of an AB antigen, and O denotes the lack of both antigens (with the presence of H antigen) [112].

#### ABH human blood group antibodies

Individuals have circulating, “natural” preformed antibodies to the missing antigen, such that individuals of type A (i.e., possessing the A antigen) have antibodies against the B antigen; those of type B have antibodies against the A antigen; and those of type O have antibodies against both the A and B antigens. These antibodies comprise IgM and IgG1/IgG3 immunoglobulins and are “natural” in the sense that they are not the result of sensitization to a previous exposure to the antigen targeted by their antibodies (e.g., due to a prior transfusion), but develop in early childhood due to exposure to similar antigens found in the environment. With respect to transplantation, consider a kidney transplant from a type A donor to a type O recipient possessing high titers of anti-A antibodies. In transplantation, these preformed anti-A antibodies circulating in the recipient’s O-type blood will encounter the A antigens expressed on the surface of epithelial and endothelial cells lining the blood and lymphatic vessels of the transplanted organ, they will recognize and bind to them thus triggering and activating the classical complement pathway and the von Willebrand factor, causing cell death and blood vessel occlusion, ultimately leading to severe graft damage, stagnation and rejection [116, 117]. Of course, in the case of transplantation, all of this needs to be avoided, a goal typically achieved to a good degree by preparation of the patient prior to transplantation surgery, mainly by removing the anti-A/anti-B antibodies (by plasma exchange) and administering immunosuppressing drugs. In fact, spurred by the substantial need for kidney (and other organ) transplants and the scarcity of organ donors, transplantation of ABO incompatible organs is now performed with some success [114, 118].

#### Solid tumor rejection therapy

**Figure 3.**
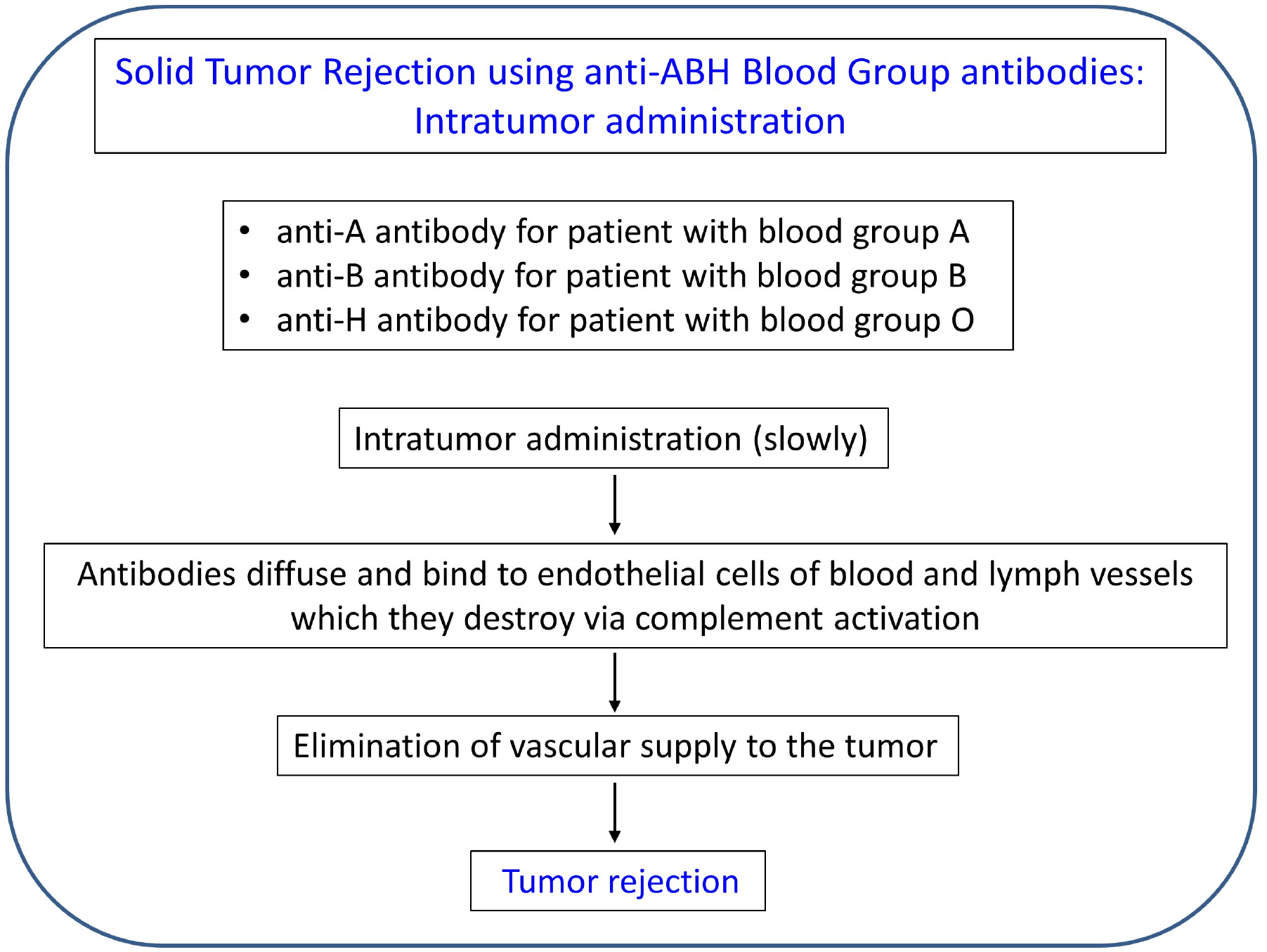
Outline of tumor rejection therapy using intra-tumor injection of anti-A/B/H antibodies. See text for details.

**Figure 4.**
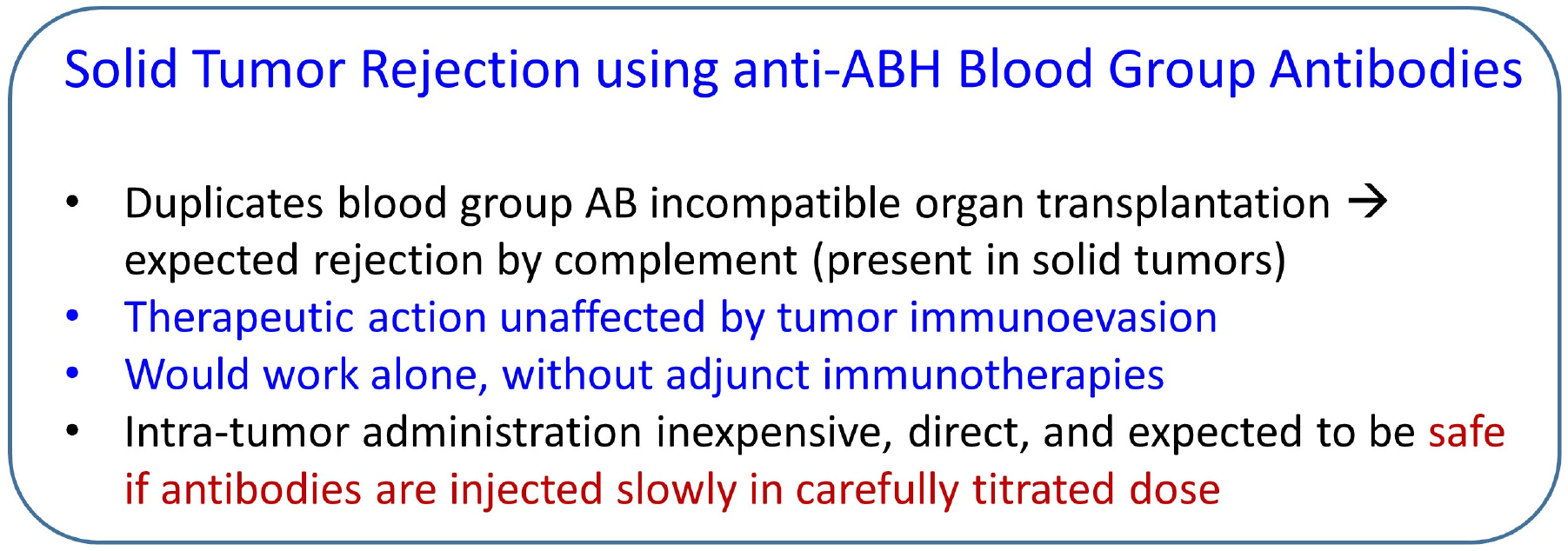
Features of tumor rejection therapy using intra-tumor injection of anti-ABH blood group antibodies.

Unlike the case of transplantation, where every effort is expended to prevent organ rejection [111, 112, 118], in the case of a solid tumor, the objective is the opposite, namely, tumor rejection. Fortunately, with this approach, the antibodies are preformed and do not need to be produced, hence the condition of the immune system in the tumor microenvironment is irrelevant. Here, the main issues concern the items to be delivered and the method and rate of delivery. The method and features of this therapy are outlined in Figures 3 and 4, respectively. With respect to the antibodies to be delivered intratumorally, there are 4 separate cases, depending on the blood type of the cancer patient. (1) For patients with type A, natural, preformed anti-A antibodies would be delivered; such antibodies exist commercially (for use in research) in a purified form or as serum from a type B donor. (2) For patients with type B, anti-B antibodies would be used. (3) For patients with type AB, either anti-A or anti-B (or a combination of both) would be used. Finally, (4) for patients of type O, anti-H antibodies would be used.

In the case of an accessible solid tumor, these antibodies can be injected intratumorally. Since this treatment can have serious adverse effects if the antibodies/antigens spread outside the tumor in substantial amounts, special care needs to be taken to inject them into the tumor precisely and at a very slow rate. Since they are very potent and act immediately, a slow infusion over a period of time is most likely to be effective; it does not have to be continuous, but for example, daily injections of small amounts would be expected to shrink the tumor gradually. Since tumors are frequently well vascularized, the antibodies should be very effective in destroying the tumor, little by little. In addition, since endothelial cells of lymphatic vessels in the tumor also express the ABH antigens, the injected anti-ABH antibodies will also destroy the lymph vessels of the tumor [119], thus limiting the spread of the tumor to adjacent lymph nodes. The main potential adverse effect is hemolysis and/or thrombosis, hence close monitoring would be essential.

Finally, attention has been drawn to developing cancer treatment based on AB incompatibility [120, 121], and results of experiments using a lentivirus to introduce the A antigen in solid breast and colorectal tumors in mice expressing anti-A antibodies have been encouraging. This approach is much more involved than the simple infusion of antibodies discussed above, but documents the positive result in solid tumor reduction effected by the concomitant presence in the tumor of the A antigen and its anti-A antibody.

## Conclusions

The evolving focus on personalized cancer therapy has already had a substantial beneficial effect on cancer treatment and survival. As might have been expected from this focus, favorable results have been observed in patients for whom treatment was successfully tailored to the specific characteristics of tumor, the patient’s genetic makeup, and up-to-date technologies in choosing, evaluating, and modifying/fine-tuning treatment (e.g., among the various kinds of immunotherapies and their combinations), as dictated by constant monitoring of changes in the tumor molecular characteristics, detecting and treating adverse effects, and assessing the status of the disease (e.g., size of the tumor, metastases, etc.) and the overall condition of the patient. Cancer immunotherapy has played a major role in this progress, yet the overall outcome of this treatment has been only moderately successful, hence the call for innovation [122]. In that respect, the incompatibility-based immunotherapies proposed above, based on HLA and ABH antigens, provide a fresh approach in cancer treatment. In addition to the intra-tumor injection of anti-ABH antibodies, a future development could employ bispecific antibodies (bsAbs) to construct antibody-drug conjugates (ADC) [123], consisting of a tumor cell-specific antibody and an anti-ABH antibody that are released at the tumor site by various mechanisms [123].

## Abbreviations

CAR: chimeric antigen receptor
CTA: cancer testis antigen
DC: dendritic cell
EV: extracellular vesicle
FDA: U.S. Food and Drug Administration
HER2: human epidermal growth factor receptor 2
HLA: human leukocyte antigen
HPV: human papilloma virus
ICI: immune checkpoint inhibitor
IFN-γ: interferon gamma
Ii: invariant chain
mAbs: monoclonal antibodies
MHC: major histocompatibility complex
ncRNA: non-coding ribonucleic acid
PET: positron emission tomography
pHLA: peptide-human leukocyte antigen complex
TAA: tumor-associated antigen
TAP: transporter associated with antigen processing
TCR: T cell receptor
TIL: tumor-infiltrating lymphocyte
TSA: tumor-specific antigen
β_2_m: β_2_-microglobulin
